# Blockade of the NLRP3/caspase-1 axis attenuates ketamine-induced hippocampus pyroptosis and cognitive impairment in neonatal rats

**DOI:** 10.1186/s12974-021-02295-9

**Published:** 2021-10-19

**Authors:** Zhiheng Zhang, Hui Bai, Xiangying Ma, Meilun Shen, Rouqian Li, Di Qiu, Siyao Li, Li Gao

**Affiliations:** 1 College of Veterinary Medicine, Northeast Agricultural University, No. 600 Changjiang Rd, Xiangfang District, Harbin, 150030 China; 2 Heilongjiang Key Laboratory for Laboratory Animals and Comparative Medicine, College of Veterinary Medicine, Northeast Agriculture University, Harbin, China

**Keywords:** Pyroptosis, Developing rats, Ketamine, NLRP3, Caspase-1

## Abstract

**Background:**

Multiple studies have revealed that repeated or long-term exposure to ketamine causes neurodegeneration and cognitive dysfunction. Pyroptosis is an inflammatory form of programmed cell death that has been linked to various neurological diseases. However, the role of NLRP3/caspase-1 axis-related pyroptosis in ketamine-induced neurotoxicity and cognitive dysfunction remains uncertain.

**Methods:**

To evaluate whether ketamine caused NLRP3/caspase1-dependent pyroptosis, flow cytometry analysis, western blotting, ELISA test, histopathological analysis, Morris water maze (MWM) test, cell viability assay, and lactate dehydrogenase release (LDH) assay were carried out on PC12 cells, HAPI cells, and 7-day-old rats. In addition, the NLRP3 inhibitor MCC950 or the caspase-1 inhibitor VX-765 was used to investigate the role of the NLRP3/caspase-1 axis in ketamine-induced neurotoxicity and cognitive dysfunction.

**Results:**

Our findings demonstrated that ketamine exposure caused cell damage and increased the levels of pyroptosis in PC12 cells, HAPI cells, and the hippocampus of neonatal rats. After continuous exposure to ketamine, targeting NLRP3 and caspase-1 with MCC950 or VX765 improved pyroptosis, reduced neuropathological damages, and alleviated cognitive dysfunction.

**Conclusion:**

NLRP3/Caspase-1 axis-dependent pyroptosis is involved in ketamine-induced neuroinflammation and cognitive dysfunction, and it provides a promising strategy to treat ketamine-related neurotoxicity.

**Supplementary Information:**

The online version contains supplementary material available at 10.1186/s12974-021-02295-9.

## Introduction

The developing brain is highly vulnerable to the adverse effects of clinically used general anesthetics (GAs), which include neuronal apoptosis [1–3], decreased synapse formation [4, 5], impaired neurogenesis [6, 7], and glial cell development [8, 9]. Preclinical and population-based retrospective studies have shown that newborns who received anesthetics during early childhood may experience long-term cognitive and behavioral impairment [10–12]. Ketamine is a non-competitive blocker of the N-methyl-D-aspartate (NMDA) receptor that is commonly used in pediatric anesthesia. Previous studies have shown that ketamine disrupts normal neurogenesis, as well as inhibits the development of nerve cells and the proliferation of neural stem cells [13, 14] in the developing brain. This causes apoptosis and neurotoxicity, which can lead to long-term learning and memory impairment [15, 16]. In addition, our previous studies revealed that the neurotoxicity of ketamine is linked to increased levels of autophagy and oxidative stress in pregnant offspring rats [17, 18]. However, the underlying mechanism by which ketamine causes neurotoxicity is still unclear. It is necessary to further investigate its mechanism and formulate strategies to identify possible specific adjunctive therapies aimed at attenuating the negative effects of ketamine.

Pyroptosis is a special form of programmed cell death characterized by the formation of plasma membrane pores, which affects membrane integrity [19]. The key feature of pyroptosis as a lytic and inflammatory process is that it relies on gasdermin proteins as the executor of cell death [20]. Activation of the classical pyroptosis pathway is triggered by inflammasomes, which are activated by acute perturbations in homeostasis (e.g., mitochondrial dysfunction, ion flux), and danger signals in the microenvironment (e.g., extracellular ATP, aberrant protein aggregates, double-stranded DNA, microbial molecules). The activation and assembly of inflammasomes promote the activation of caspase-1. Subsequently, the inflammatory caspase promotes the cleavage of the gasdermin family proteins, which induces pore formation in the plasma membrane, resulting in pyroptosis-induced lytic cell death and the release of cytokines [20, 21]. Recent studies have linked pyroptosis to neurodegenerative and neuroinflammatory disorders, such as sepsis [22], traumatic brain injury [23], stroke [24], and Alzheimer’s disease [25]. Although several studies have shown that continuous exposure to anesthetics activates the NLRP3 inflammasome and caspase-1 [26–30], whether NLRP3/caspase-1 axis is involved in ketamine-induced cognitive and behavioral dysfunction remains largely unknown.

In this study, we hypothesize that NLRP3/caspase-1 axis-dependent pyroptosis is involved in the mechanism of ketamine-induced cognitive and behavioral dysfunction in neonatal rats and that inhibiting the NLRP3 inflammasome with MCC950 and caspase-1 with VX765 may ameliorate ketamine-induced hippocampus pyroptosis, as well as cognitive and behavioral dysfunction. To validate this hypothesis, we investigated the role of the NLRP3/caspase-1 axis in ketamine-induced neuroinflammation and cognitive dysfunction in vitro and in vivo, as well as the protective effects of MCC950 or VX765.

## Materials and methods

### Animals

Seven-day-old Sprague Dawley rats (half male and half female) were purchased from the Animal Center of Harbin Medical University (Harbin, China). All experimental procedures and protocols were carried out in accordance with the guidelines of the Experimental Animal Ethics Committee of Northeast Agricultural University.

### Ketamine-induced neurotoxicity model and drugs treatment

Ketamine-induced 7-day-old neurotoxicity rats were produced according to a previously reported method [31, 32]. All rats were randomized into six groups:

1. **Con**, the normal saline control group that received normal saline only.
2. **Ket**, continuously exposed to ketamine.
3. **MCC950**, received 10 mg/kg MCC950 (MedChemExpress, China).
4. **MCC950 + Ket,** received 10 mg/kg MCC950 before ketamine injection.
5. **VX765**, received 25 mg/kg VX765 (MedChemExpress, China).
6. **VX765 + Ket**, received 25 mg/kg VX765 before ketamine injection.

Each group received five doses of ketamine (20 mg/kg) or normal saline every 90 min (*n* = 25). The rats were administered MCC950, VX765, or normal saline intraperitoneally 30 min before ketamine treatment. All drugs were diluted in normal saline, and the volume of each injection was 0.1 mL. During the experiment, the rats were placed in an incubator with a 1.5 L/min O_2_ flow rate to avoid hypoxia and hypothermia. Samples were collected 90 min after the last dose of ketamine: nine rats were euthanized for protein analysis, six rats were perfused with 4% paraformaldehyde for Nissl staining and Terminal deoxynucleotidyl transferase-mediated dUTP-biotin nick end labeling (TUNEL) analysis, and ten rats were reared for 60 days for the Morris water maze experiment.

### Nissl staining and TUNEL analysis

After being embedded in paraffin, the brain tissue samples were cut into 5-μm-thick slices, and Nissl staining was performed. All sections were observed under 40 × to 200 × magnification and nine 10^4^ μm^2^ visual fields from three rats (three fields per rats/sections) were randomly selected at 400 × magnification in the Cornu Ammonis 1 (CA1), CA3, and Dentate Gyrus (DG) of the hippocampus for quantitative cells assay.

For TUNEL staining, sections (*n* = 3) were dewaxed using xylene, permeabilized with proteinase K solution (Beyotime, Shanghai, China), incubated with terminal deoxynucleotidyl transferase (TdT) and dUTP (Beyotime) at 37 ℃, followed by nuclei staining with DAPI (Beyotime). The stained sections were visualized under a fluorescence microscope at 400× magnification.

### Cell culture and drug treatment

PC12 (rat pheochromocytoma of adrenal medulla cell line) and HAPI (Highly Aggressive Proliferating Immortalized) cells were cultured in DMEM (Dulbecco's Modified Eagle Medium, Meilunbio, Dalian, China) medium containing 10% fetal bovine serum (FBS, Biological Industries, Israel), penicillin, and streptomycin at 37℃ with 5% CO_2_ and 95% air. The following were the experimental groups:

1. **Con**, the control group, where cells were cultured in complete medium without any treatment for 24 h.
2. **Ket**, cells were cultured in complete medium with 1.5 mM ketamine for 24 h.
3. **MCC950**, cells were cultured in complete medium with 10 μM MCC950 for 24 h.
4. **MCC950 + Ket**, cells were pretreated with 10 μM MCC950 for 30 min before being cultured in complete medium with 1.5 mM ketamine for 24 h.
5. **VX765**, the cells were cultured in complete medium with 10 μM VX765 for 24 h.
6. **VX765 + Ket**, The cells were pretreated with 50 μM VX765 for 30 min before being cultured in complete medium with 1.5 mM ketamine for 24 h.

### Cell viability assay

Cell viability was determined using the cell counting kit (CCK)-8 solution (DOJINDO, Japan). To determine the effects of MCC950 and VX765 on ketamine-induced cell death, the cells were cultured in complete medium with different treatments, and 10 μL CCK-8 solution was added to 100 μL complete medium for 2 h at 37 ℃ in each well. The absorbance of each well was measured at 450 nm using a microplate reader (BioTek, Thermo Fisher Scientific, USA).

### Lactate dehydrogenase release assay

After the cells were treated with different drugs, the culture supernatants were collected. The levels of released LDH in the supernatants were measured using the LDH Cytotoxicity Assay Kit (Beyotime) according to the manufacturer’s instructions. The absorbance at 490 nm was measured with a microplate reader.

### Caspase-1/PI activity assay

Pyroptosis in each group was measured using the FAM-YVAD-FMK/PI detection kit (Cat^#^97, ImmunoChemistry, USA). Active caspase-1 was labeled with FAM-YVAD-FMK, membrane pore formation was labeled with propidium iodide (PI), and pyroptosis was defined as being double positive for both caspase-1 and PI. A total of 1.0 × 10^5^–1.0 × 10^6^ cells (one sample was taken from every three bottles of cells) in suspension was incubated with FAM-YVAD-FMK for 1 h at 37 ℃ (protected from light). After centrifugation, the supernatant was removed by aspiration and the cell pellet was washed with wash buffer, followed by a 15 min incubation with 5μL PI. Finally, the samples were analyzed using a BD FACSAria flow cytometer (Becton Dickinson and Company, USA).

### Western blot analysis

The hippocampus and cells were lysed in RIPA buffer (Beyotime, Shanghai, China). Total protein concentrations were measured using the BCA protein assay kit (Thermo Fisher Scientific). Lysed protein was separated using a 10–12% SDS-PAGE gel and transferred to nitrocellulose membranes. The membranes were blocked in 5% skim milk and incubated at 4 ℃ overnight with primary antibodies against caspase-1 (ABclonal, A0964, 1: 1000), NLRP3 (ABclonal, A14223, 1: 1000), ASC (ABclonal, A16672, 1: 1000), GSDMD (ABclonal, A10164, 1: 1000), or GAPDH (ABclonal, A19056, 1: 1000), respectively. This was followed by incubation with an HRP-conjugated secondary antibody for 1.5 h at room temperature. The densities of the protein bands were detected using an ECL kit, imaged using a Tanon5200 Gel Imaging System (Tanon, China), and analyzed using the Image J software. One sample was taken from every three tissues or three bottles of cells (PC12 or HAPI), and measurements were performed in triplicate.

### ELISA

Concentrations of IL-1β and IL-18 in the hippocampus samples and cell supernatants were measured using specific rat enzyme-linked immunosorbent assay (ELISA) kits (Jiangsu Jingmei Biological Technology Co., Ltd, Jiangsu, China) according to the manufacturer’s instructions.

### Morris water maze (MWM) test

The Morris water maze (MWM) test was used to assess the ability of spatial learning and memory in rats 60 days after birth, with ten rats per group [33, 34]. In brief, a transparent platform (12 cm diameter) was submerged in a circular pool (diameter: 150 cm, depth: 60 cm) and filled with warm (25 ± 1 °C) opaque water. The MWM test consisted of two phases: 5 days of training and the sixth day with exploratory trials. During the training phase, each rat was released from four random locations (NW, NW, SE, and SW) in the pool to find the hidden platform. The experiment was terminated when the rats reached the hiding platform. If the rats failed to find the platform within 90 s, they were guided to stay in place for 15 s, and the delay was recorded as 90 s. The rats were trained four times a day. On day 6, the hidden platform was removed for the spatial probe test. Each rat was allowed to swim for 90 s to test their memory. The swimming speed (cm/s), escape latency, swimming path tracks, and the number of entries into the platform quadrant zone for each day during spatial training of MWM were recorded and analyzed with the ANYmaze video tracking system (Stoelting Co., IL, USA).

### Statistical analysis

Data were presented as mean ± standard deviation (SD), and each experiment was performed at least in triplicate. All statistical analyses were performed using the SPSS software (Version 22.0). Data were analyzed using one-way ANOVA followed by Tukey’s post-hoc test or by Dunnett’s test if data were not normally distributed. Behavioral data were analyzed using repeated two-way ANOVA followed by the LSD test. *P* < 0.05 was considered statistically significant.

## Results

### The effect of MCC950 and VX765 on the ketamine-induced hippocampal loss of nerve cells in neonatal rats

**Fig. 1:**
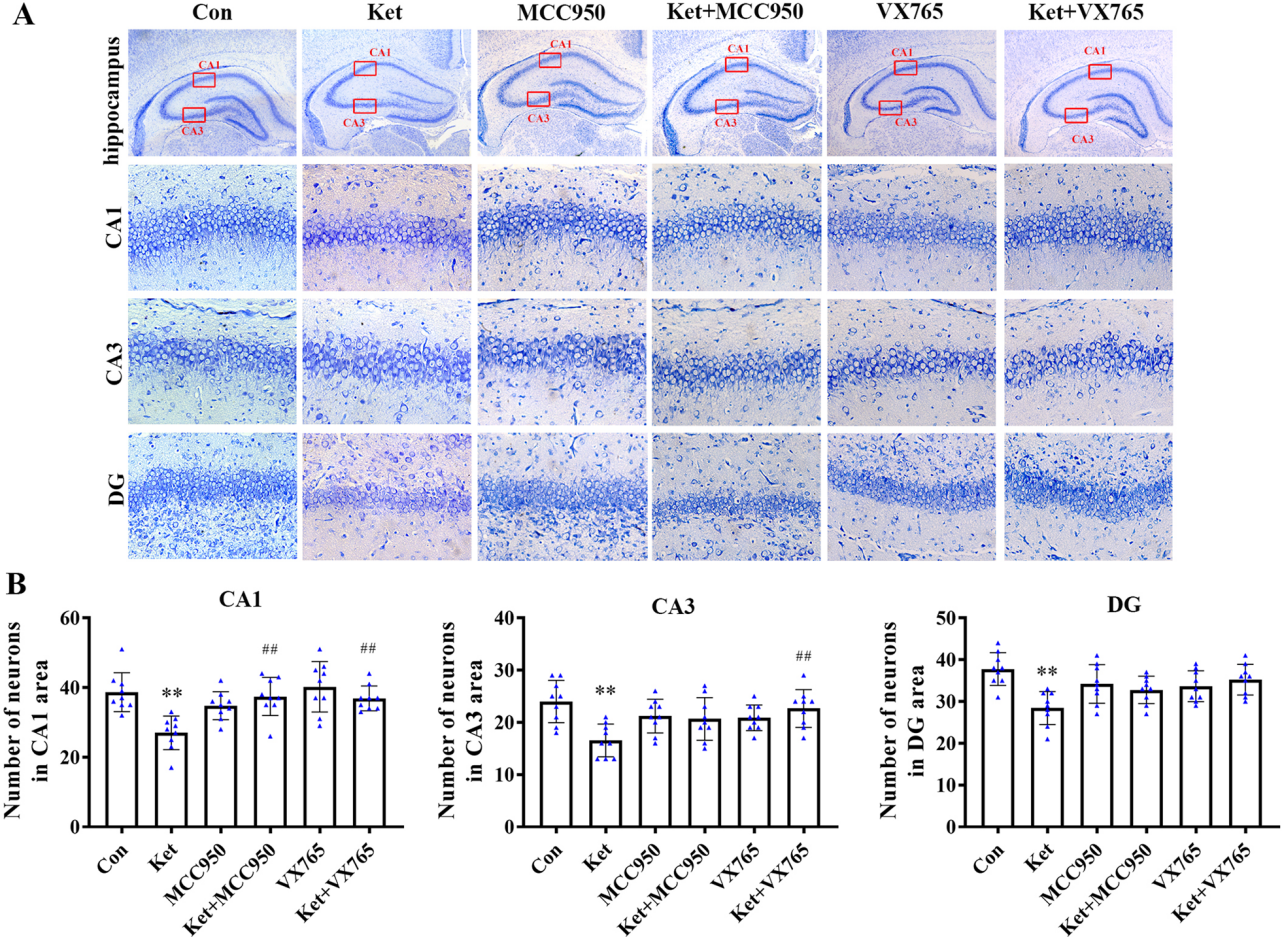
MCC950 or VX765 attenuates the loss of nerve cells in the hippocampus of neonatal rat exposure to ketamine. **A** Nissl staining hippocampus (CA1, CA3, and DG region). Hippocampus was viewed at a magnification of 40×; the others, at 400×. **B** Number of cells in CA1, CA3, DG area. Values are expressed as mean ± SD. *P < 0.05, **P < 0.01, compared with group C; ^#^ *P* < 0.05, ^##^*P* < 0.01, compared with group Ket. Con, control; Ket, ketamine. CA1, Cornu Ammonis 1, CA3, Cornu Ammonis 3. DG, Gentate Gyrus

To detect the neuroprotective effect of MCC950 or VX765 on nerve cell damage caused by ketamine, Nissl staining was performed on tissue sections. The hippocampus plays a key role in spatial learning and episodic memory, with the Cortical Area 1 (CA1), CA3, and Dentate Gyrus (DG) regions involved in spatial navigation and episodic memory formation. Based on this, quantitative cell assay was performed in the CA1, CA3, and DG regions of the hippocampus. The results showed that pre-administration of MCC950 and VX765 decreased the loss and disorder of nerve cells caused by continuous ketamine exposure in the hippocampus (CA1, CA3, and DG regions) of neonatal rats (Fig. 1A and B). This finding indicated that MCC950 or VX765 protected hippocampal nerve cells from ketamine-induced morphological damage and that the NLRP3/Caspase-1 axis may be involved in the loss of hippocampal nerve cells caused by ketamine.

### Ketamine induces hippocampal pyroptosis by activating the NLRP3/Caspase-1 axis in neonatal rats

**Fig. 2:**
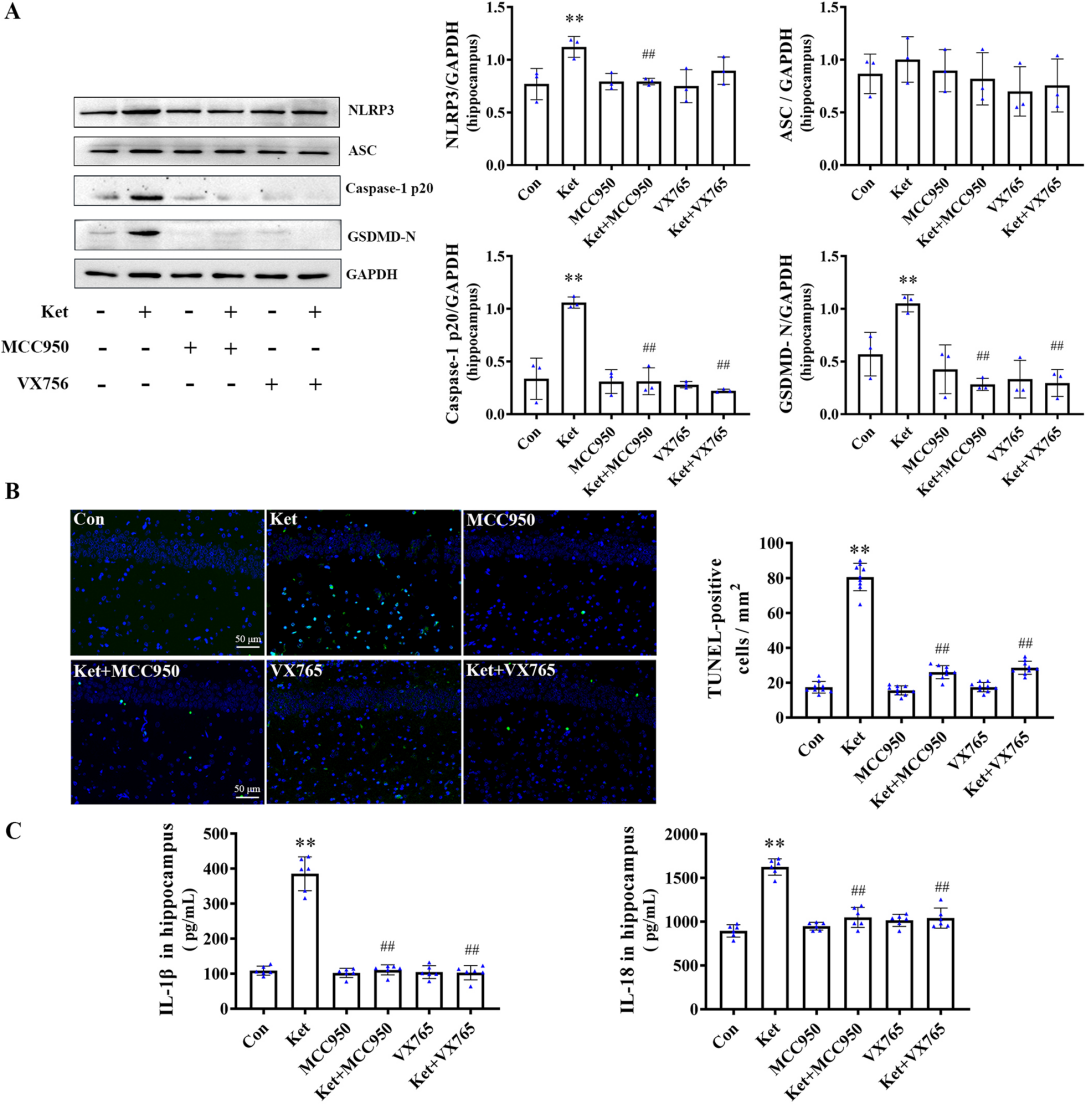
Effect of inhibition of NLRP3/Caspase-1 axis on ketamine-induced pyroptosis in hippocampus of neonatal rats. **A** Protein bands and quantitative analysis of NLRP3-3, ASC, cleaved Caspase-1, and cleaved GSDMD (*n* = 3). **B** TUNEL staining in hippocampus and the number of positive cell, Scale bar 50 μm. **C** Cytokines levels of IL-1β and IL-18 in hippocampus (*n* = 6). Values are expressed as the mean ± SD. *P < 0.05, **P < 0.01, compared with group C; ^#^ *P* < 0.05, ^##^*P* < 0.01, compared with group Ket. Con, control; Ket, ketamine

To further investigate whether the NLRP3/Caspase-1 axis is involved in ketamine-induced pyroptosis, a pyroptosis-related test in the hippocampus was performed. Western blot analysis was used to determine the protein expression of NLRP3, ASC, cleaved caspase-1, and cleaved GSDMD (GSDMD-N) in the hippocampus of 7-day-old rats. ELISA analysis was used to detect the levels of IL-1β and IL-18, and a TUNEL assay was used to determine the number of cell deaths. The results revealed that the protein levels of NLRP3, cleaved caspase-1, and cleaved GSDMD (Fig. 2A), the number of TUNEL-positive cells (Fig. 2B), and the levels of IL-1β and IL-18 (Fig. 2C) increased after continued ketamine exposure. Notably, pretreatment with MCC950 or VX765 reduced the observed increases (Fig. 1A–C). There was no significant change in ASC for each group. These findings indicated that the NLRP3/Caspase-1 axis is involved in hippocampal pyroptosis induced by ketamine.

### The effects of NLRP3/Caspase-1 Inflammasome inhibitors on cell viability induced by ketamine on PC12 or HAPI cells

**Fig. 3:**
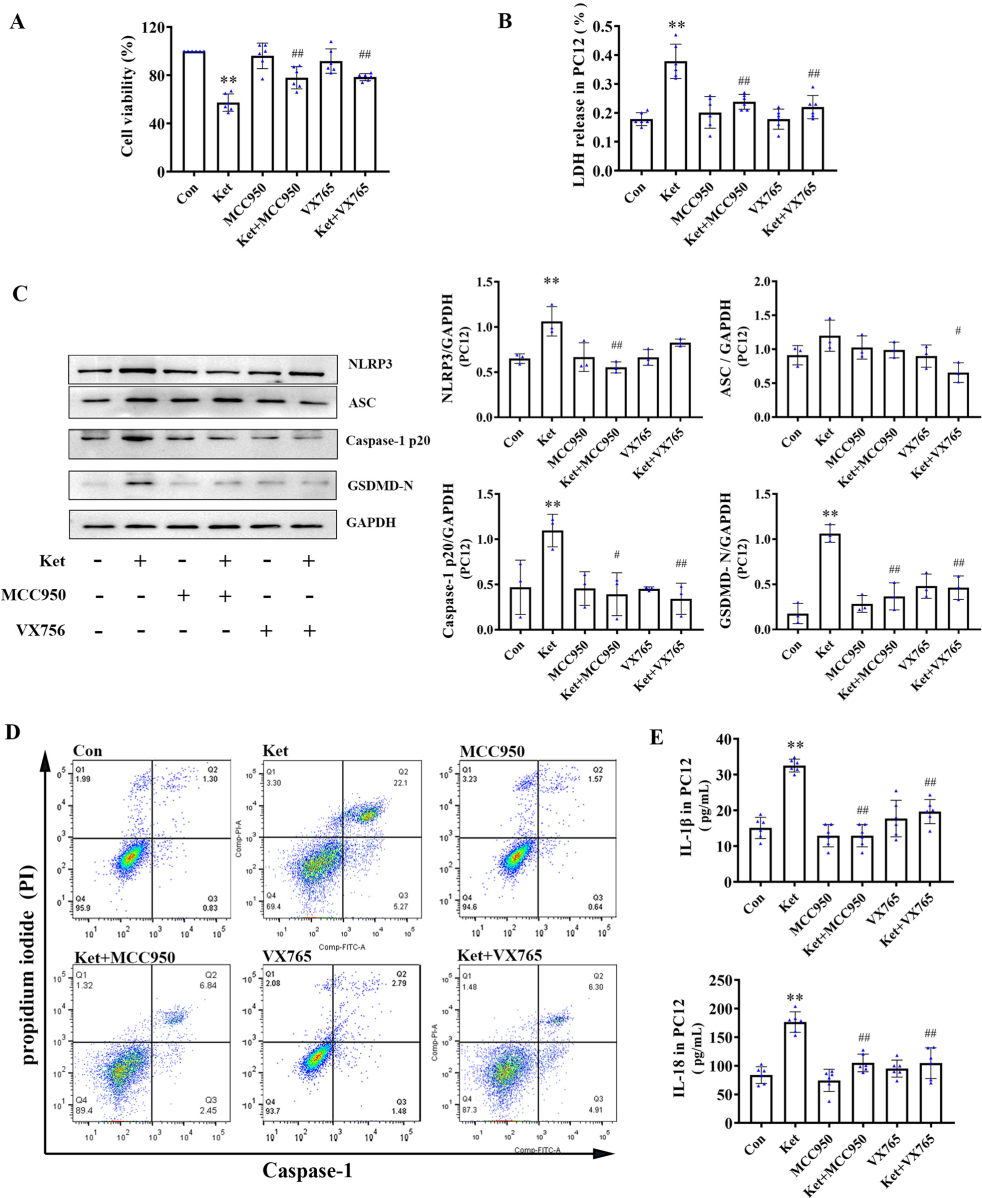
Ketamine-induced pyroptosis in PC12 cells is NLRP3/Caspase-1 inflammasome-dependent. **A** Cell activity analysis in PC12 cells. **B** LDH release test. **C** Western blotting analysis of NLRP3-3, ASC, cleaved Caspase-1, and cleaved GSDMD (*n* = 3) **D** Effects of NLRP3/Caspase-1 Inflammasome inhibitors on ketamine-induced double-positivity for PI and activated caspase-1 flow cytometry analysis. **E** The cytokines levels of IL-1β and IL-18 in PC12 cells (*n* = 6). Values are expressed as the mean ± SD. *P < 0.05, **P < 0.01, compared with group C; ^#^ *P* < 0.05, ^##^*P* < 0.01, compared with group Ket. Con, control; Ket, ketamine

**Fig. 4:**
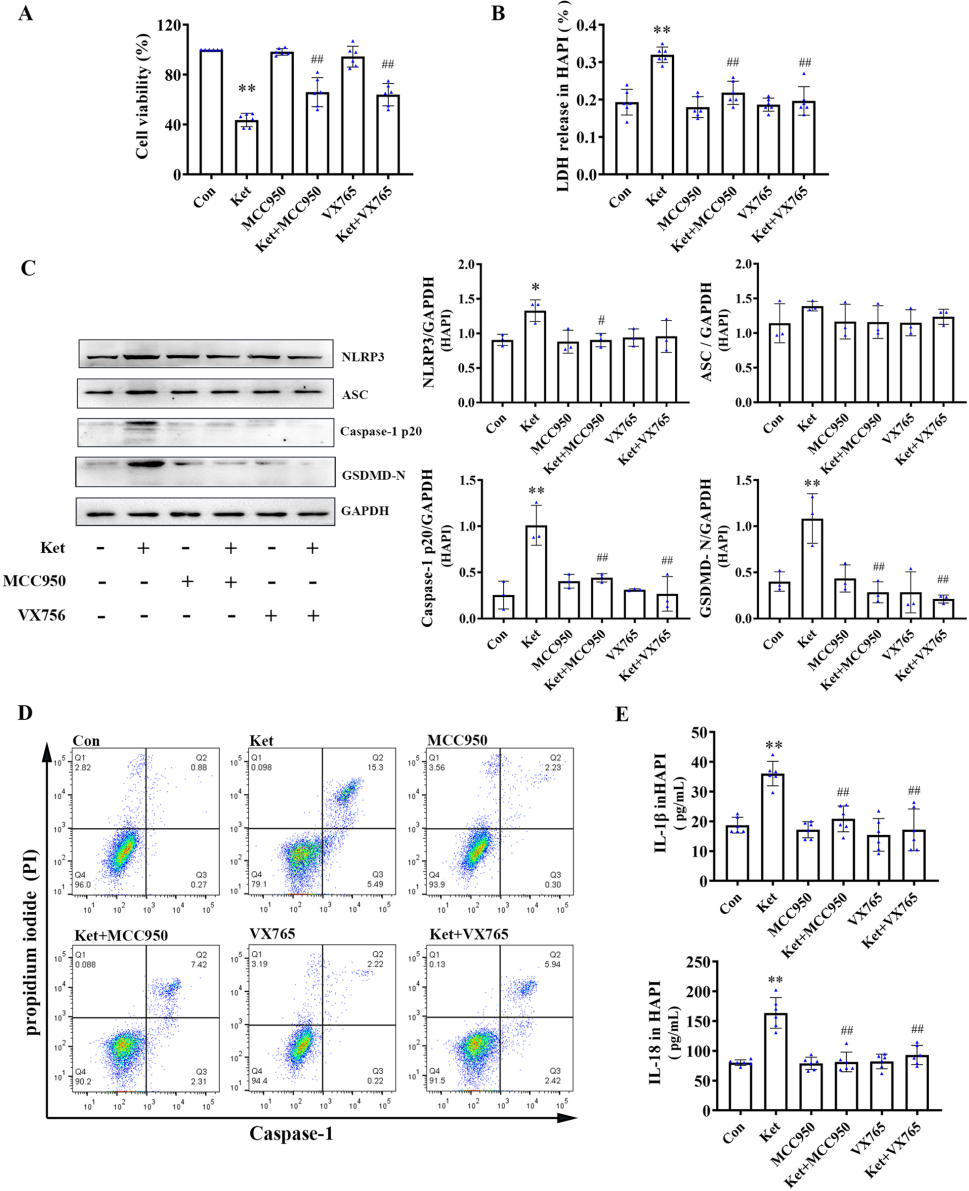
Ketamine-induced pyroptosis in HAPI cells is NLRP3/Caspase-1 inflammasome-dependent. **A** Cell activity analysis in HAPI cells. **B** LDH release Assay. **C** Western blotting analysis of NLRP3-3, ASC, cleaved Caspase-1, and cleaved GSDMD (*n* = 3) **D** Flow cytometry analysis for activated Caspase-1/PI. **E** The cytokines levels of IL-1β and IL-18 in HAPI cells (*n* = 6). Values are expressed as the mean ± SD. *P < 0.05, **P < 0.01, compared with group C; ^#^ *P* < 0.05, ^##^*P* < 0.01, compared with group Ket. Con, control; Ket, ketamine

To verify the connection between continuous ketamine exposure to and pyroptosis in vitro, HAPI and PC12 cells were selected for cell viability analysis. In PC12 cells, NLRP3/Caspase-1 inhibitors (MCC950 or VX765) ameliorated cell death after exposure to ketamine for 24 h (Fig. 3A). In addition, similar results were observed in HAPI cells treated with MCC950 or VX765 (Fig. 4A).

### NLRP3/Caspase-1 inflammasome inhibitors block the pyroptosis caused by ketamine in PC12 cells

To examine the role of the NLRP3/Caspase-1 axis in ketamine-induced pyroptosis in PC12 cells, the activation of NLRP3/Caspase-1 inflammasome was inhibited with MCC950 or VX765, respectively. As shown in Fig. 3B, pretreatment with MCC950 or VX765 inhibited the release of LDH caused by ketamine. The protein expression of NLRP3, cleaved caspase-1, and cleaved GSDMD, as well as the levels of inflammatory cytokines (IL-1β and IL-18) increased after ketamine treatment for 24 h, indicating that there was an increase in the level of pyroptosis in PC12 cells (Fig. 3C and E). Both MCC950 and VX765 reduced the effect of ketamine in PC12 cells. In addition, flow cytometry (FCM) was conducted to determine caspase-1 activation and membrane pore formation. The amount of caspase-1/PI double-positive cells induced by ketamine was decreased by MCC950 or VX765 (Fig. 3D). These findings indicated that the NLRP3/Caspase-1 axis was involved in ketamine-induced pyroptosis in PC12 cells.

### NLRP3/Caspase-1 Inflammasome inhibitors down-regulate ketamine-induced pyroptosis in HAPI cells

Glial cells account for around 70% of the total number of cells in the central nervous system (CNS) and play an important role in neuromodulation, neurotrophic, and neuro-immunity. The HAPI cell line is widely used in many studies involving glial cells [35, 36]. Here, the effects of MCC950 and VX7650 on pyroptosis by continuous ketamine exposure in HAPI cells were evaluated. After ketamine exposure, pretreatment with MCC950 or VX765 significantly decreased the level of LDH release in HAPI cells (Fig. 4B). In addition, the protein levels of NLRP3, cleaved Caspase-1, and cleaved GSDMD, and cytokine (IL-1β and IL-18) levels increased after HAPI cells were treated with ketamine for 24 h (Fig. 4C and E). Both MCC950 and VX765 reduced the effect of ketamine in HAPI cells. Interestingly, caspase-1/PI staining revealed that both MCC950 and VX765 reduced the number of activated caspase-1/PI double-positive cells induced by ketamine in HAPI cells (Fig. 4D). These results suggested that NLRP3/Caspase-1 axis was involved in ketamine-induced pyroptosis in HAPI cells.

### Blockade of NLRP3/Caspase-1 axis alleviates cognitive and behavioral dysfunction caused by ketamine in neonatal rats

**Fig. 5:**
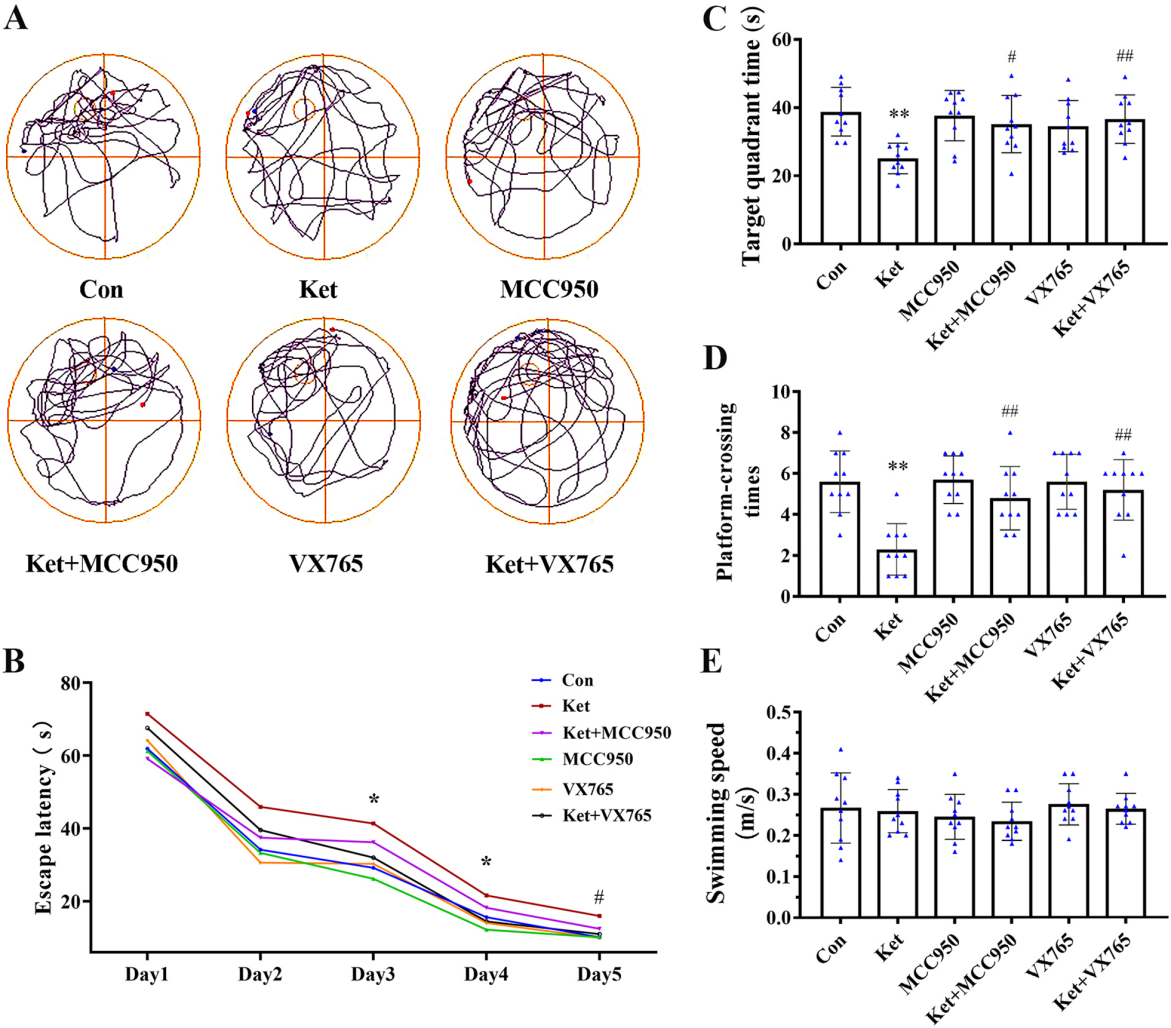
Blockade of NLRP3/Caspase-1 axis prevents ketamine-induced cognitive and behavioral dysfunction in neonatal rats. **A** Swimming path of the rats. **B** Escape latency to reach the platform. **C** Time spent in the target quadrant. **D** Number of hidden platform traversal times. **E** Mean swimming speed. Values are expressed as the mean ± SD (*n* = 10). *P < 0.05, **P < 0.01, compared with group C; ^#^*P* < 0.05, ^##^*P* < 0.01, compared with group Ket. Con, control; Ket, ketamine

To determine the effect of the NLRP3/Caspase-1 axis on ketamine-induced cognitive dysfunction in neonatal rats, MCC950 and VX765 were used and an MWM experiment was performed. In the probe trial test, rats exposed to ketamine displayed a long escape latency (Fig. 5B) in the training phase, as well as a decreased target quadrant time (Fig. 5C) and platform-crossing times (Fig. 5D). Interestingly, pretreatment with MCC950 or VX765 significantly improved the performances of the rats in the WMW tests. There were no significant changes in average swimming speed in each group (Fig. 5E). In our study, sex-specific differences did not affect the results of behavioral experiments in young rats (Additional file 1: Fig. S1). These findings indicated that NLRP3/Caspase-1 axis was related to ketamine-induced cognitive and behavioral dysfunction, and that intraperitoneal administration of MCC950 or VX765 was effective in treating ketamine-induced cognitive and behavioral dysfunction.

## Discussion

This study provided novel insights into the role of the inflammasome pathway in the neurotoxicity mechanism and treatment induced by ketamine. Our findings revealed the involvement of the NLRP3/caspase-1 axis in ketamine-induced neuroinflammation and cognitive dysfunction and that MCC950 or VX765 provided neuroprotection against this.

The developing brain is particularly vulnerable to ketamine neurotoxicity compared to the mature adult brain [37]. In rodents, the critical period of brain development is during the first 1–3 weeks of life; whereas in humans, this period begins during the last trimester of gestation and lasts until 2–3 years of age [37, 38]. Any interference in brain development during these critical periods is likely to result in long-term brain injury, and ultimately lead to neuro-related behavioral dysfunction [39]. Repeated exposure to general anesthetics before the age of three is believed to be harmful to the developing brain [40]. Ketamine is commonly used as an anesthetic and analgesic in pediatrics and obstetrics. High doses or continuous ketamine exposure have been shown to cause long-term-lasting cognitive dysfunction in rodents and primates [3, 11, 15, 16, 31]. In this study, a loss of hippocampal nerve cells and long-term cognitive dysfunction were observed in 7-day-old rats after ketamine exposure, which was consistent with previous studies. It has been shown that continuous exposure to ketamine may compensatively up-regulate the expression of NMDA receptor subunits, causing endogenous glutamate to activate NMDA receptors, ultimately increasing intracellular Ca^2+^ influx [41, 42]. A prolonged presence of high intracellular Ca^2+^ concentrations could be a trigger for ketamine-induced neurotoxicity [43]. Recent studies have demonstrated that Ca^2+^ is a key molecular regulator of NLRP3 inflammasomes, and an increase in cytoplasmic Ca^2+^ promotes the assembly and activation of NLRP3 inflammasomes [44, 45]. High doses of ketamine caused neurotoxicity in the mouse hippocampus, as well as the activation of NLRP3 and cleavage of caspase-1 [28], implying that the neurotoxic effect of ketamine may be linked to NLRP3/Caspase-1-related pyroptosis.

The NLRP3 inflammasome is a critical component of inflammasomes that forms a complex with the apoptosis-associated speck-like protein (ASC) and the precursor form of caspase-1, resulting in the cleavage of caspase-1 and the maturation and secretion of IL-1β and IL-18 [46, 47]. Similar to previous studies, we demonstrated that clinical doses of ketamine (20 mg/kg) induced pyroptosis in the hippocampus of 7-day-old rats, presenting several signs of pyroptosis, including increased protein expression of NLRP3, cleaved caspase-1, and inflammatory cytokine (IL-1β and IL-18) levels. In addition, the activated NLRP3 inflammasome-caspase-1 can cleave GSDMD to yield the GSDMD-N domain. GSDMD-N, as the key executor of cell pyroptosis, eventually forms pores on the lipid membrane and induces cell lysis [20, 21, 48]. Furthermore, we found that the cleaved GSDMD was upregulated in the hippocampus of 7-day-old rats after ketamine exposure. Meanwhile, we also found the same effect of ketamine on pyroptosis in different cell types (HAPI or PC12 cells). These preliminary findings indicated that the activation of the NLRP3/Caspase-1 axis could be linked to ketamine-induced pyroptosis and neuroinflammation. In addition, the activation of this axis may be a condition of ketamine-induced neurotoxicity, and alleviating this process could aid in the development of effective treatment strategies.

NLRP3/caspase-1 axis activation and pyroptosis have been shown to play vital roles in neuroinflammation and neuronal damage in various neurological disease models [22–24, 28, 29, 49]. Based on previous research and our preliminary findings, we hypothesize that NLRP3/caspase-1axis related-pyroptosis plays an important role in ketamine-induced hippocampus damage and cognitive dysfunction. This was validated by the effects of MCC950 and VX765 that were observed in vitro and in vivo. MCC950 is a highly potent inhibitor that specifically targets and inhibits the NLRP3 inflammasome [50], while VX765 inhibits caspase-1 activation, as well as reduces IL-1β and IL-18 secretion [51]. Both MCC950 and VX765 have been shown to have neuroprotective properties in multiple neurological disorders [52–55]. In this study, we discovered that MCC950 and VX765 had no obvious effects on the anesthetic and analgesic properties of ketamine (Additional file 1: Fig. S2). The effects of inhibiting the NLRP3/caspase-1 axis with MCC950 and VX765 on hippocampal damage caused by continuous exposure to ketamine in young rats can be summarized as follows: (1) MCC950 or VX765 effectively alleviated the loss of hippocampal nerve cells (CA1, CA3, and DG areas); (2) MCC950 or VX765 downregulated the expression of pyroptosis-related indicators and the secretion of inflammatory cytokines in vivo and in vitro. Taken together, these findings revealed that activation of the NLRP3/Caspase-1 axis was involved in hippocampal neuronal damage and neuroinflammation induced by ketamine.

Both MCC950 and VX765 can freely cross the blood–brain barrier [56, 57]. MCC950 has been shown to down-regulate the levels of pyroptosis in the hippocampus of diabetic db/db mice and improve cognitive dysfunction [58]. On the other hand, VX765 was reported to alleviate cognitive damage and neuropathology in an Alzheimer's disease mouse model [57]. In this study, we investigated whether hippocampal damage induced by the activation of the NLPR3/Caspase-1 axis was related to cognitive dysfunction in this model. Pretreatment with either 10 mg/kg MCC950 or 25 mg/kg VX765 was effective in ameliorating cognitive dysfunction caused by ketamine exposure in 7-day-old rats. In summary, our findings revealed that NLRP3/Caspase-1 axis-dependent pyroptosis had a negative role in the pathogenesis of ketamine-induced cognitive dysfunction in neonatal rats and that MCC950 or VX765 could be used as a potential therapeutic compound for the treatment of ketamine-induced neurotoxicity. Future studies investigating the detailed mechanism by which ketamine induces the activation of the NLRP3/Caspase-1 axis are required.

## Conclusion

This was the first study, at least to our knowledge, that showed the involvement of NLRP3/Caspase-1 axis-dependent pyroptosis in ketamine-induced neurotoxicity, as well as cognitive and behavioral dysfunction. This study demonstrated that inhibition of NLRP3/Caspase-1 with MCC950 or VX765 could be effective in treating cognitive dysfunction and neuroinflammation caused by ketamine in developing rats.

## Supplementary Information

**Additional file 1: Fig. S1.** Effects of sex on blocking the NLRP3/Caspase-1 axis to prevent ketamine-induced cognitive dysfunction in neonatal rats. **Fig. S2.** Effect of VX765 and MCC950 on anesthetic effect of ketamine.

## Data Availability

All data generated or analyzed during this study are included in the published article and its additional files.

## Abbreviations

GAs: General anaesthetics
NMDA: N-Methyl-d-aspartate
CCK-8: Cell Counting Kit-8
CNS: Central nervous system
ELISA: Enzyme-Linked Immunosorbent Assay
FBS: Fetal bovine serum
NLRP3: Nod-like receptor family protein 3
IL-1β: Interleukin 1β
IL- 18: Interleukin 18
ASC: Apoptosis-associated speck-like protein
GSDMD: Gasdermin D
ANOVA: Analysis of variance
SD: Standard deviation
GAPDH: Glyceraldehyde-3-phosphate dehydrogenase; MWM: Morris water maze
