# Supplementary material for: Blockade of the NLRP3/caspase-1 axis attenuates ketamine-induced hippocampus pyroptosis and cognitive impairment in neonatal rats
Source: J Neuroinflammation. 2021 Oct 19;18:239. doi: 10.1186/s12974-021-02295-9 (PMC8527745; doi:10.1186/s12974-021-02295-9)
Supplement: Supplementary file 1 — Additional file 1: Fig. S1. Effects of sex on blocking the NLRP3/Caspase-1 axis to prevent ketamine-induced cognitive dysfunction in neonatal rats. Fig. S2. Effect of VX765 and MCC950 on anesthetic effect of ketamine. [file 12974_2021_2295_MOESM1_ESM.docx]

**Additional files**


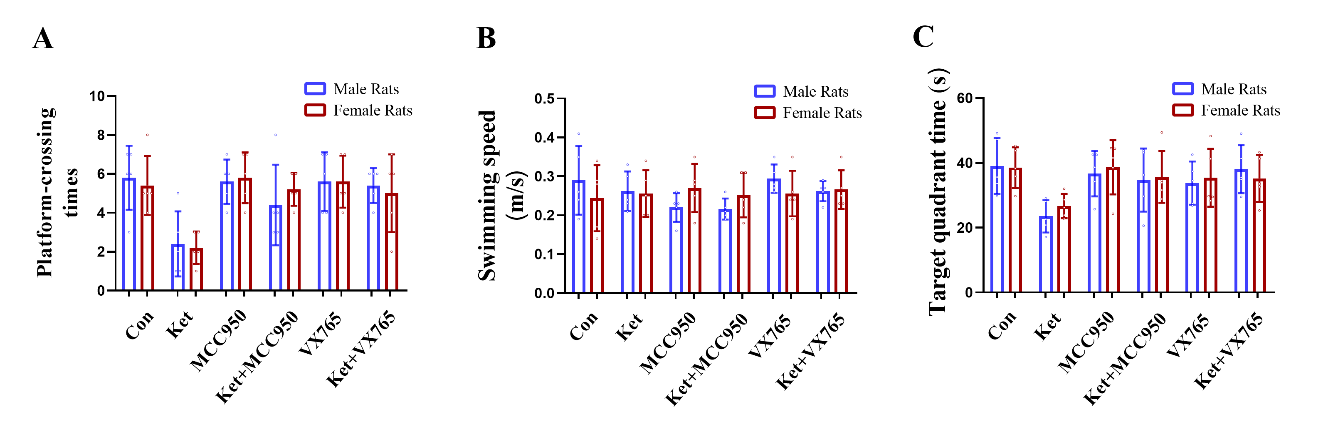


**Additional file 1: Fig. S1.** Effects of sex on blocking the NLRP3/Caspase-1 axis to prevent ketamine-induced cognitive dysfunction in neonatal rats. (A) Number of hidden platform traversal times. (B) Mean swimming speed. (C) Time spent in the target quadrant. Values are expressed as the mean ± SD (n = 5). **P* < 0.05, ***P* < 0.01, compared with Male Rats; C, control; Ket, ketamine.


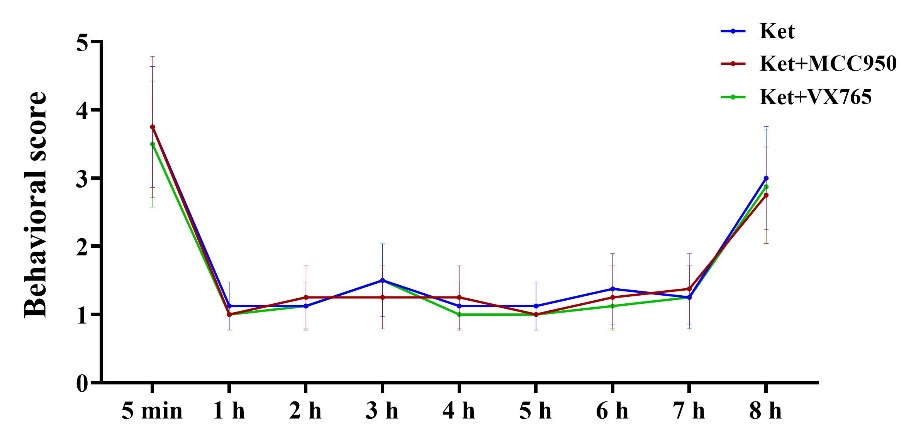


**Additional file 2: Fig. S2.** Effect of VX765 and MCC950 on anesthetic effect of ketamine. The quality of the anaesthesia was assessed by toe-web clamping and was evaluated using a scoring system [1, 2] with one indicating no response and five a marked response: 0 represents an animal that can walk without ataxia with smooth induction/recovery; 1 indicates lack of any response to toe-web clamping, slow and deep respirations; 2 indicates slight rise in muscle tone in response to toe-web clamping; considered to be a superficial reflex response; 3 represents slight withdrawal response confined to the pinched limb; considered to be a stronger reflex response and related with ‘lighter’ anaesthesia; 4 indicates obvious response in the tested leg; occasional movement elsewhere in response to toe-web clamping; 5 represents rapid withdrawal of leg and spontaneous movement of other limbs in response to toe-web clamping. Values are expressed as the mean ± SD (n = 8). **P* < 0.05, ***P* < 0.01, compared with Ket at the same time point; Ket, ketamine.

1. Jang H, Choi H, Lee S, Jang K, Lee M: **Evaluation of the anaesthetic effects of medetomidine and ketamine in rats and their reversal with atipamezole.** *Vet Anaesth Analg* 2009, **36:**319-327.

2. Antunes L, Roughan J, Flecknell P: **Evaluation of auditory evoked potentials to predict depth of anaesthesia during fentanyl/fluanisone-midazolam anaesthesia in rats.** *Vet Anaesth Analg* 2001, **28:**196-203.
